# Dietary Anthocyanin Intake and Risk of Metabolic Dysfunction-Associated Steatotic Liver Disease: Results from the NUTRIHEP Study

**DOI:** 10.3390/antiox15070802

**Published:** 2026-06-26

**Authors:** Rossella Tatoli, Rossella Donghia, Gianluigi Casimo, Pasqua Letizia Pesole, Caterina Bonfiglio

**Affiliations:** 1 Unit of Data Science, National Institute of Gastroenterology—IRCCS “Saverio de Bellis”, Castellana Grotte, 70013 Bari, Italy; rossella.donghia@irccsdebellis.it (R.D.); gianluigi.casimo@irccsdebellis.it (G.C.); catia.bonfiglio@irccsdebellis.it (C.B.); 2 Core Facility Biobank, National Institute of Gastroenterology “Saverio de Bellis”, IRCCS Hospital, Castellana Grotte, 70013 Bari, Italy; letizia.pesole@irccsdebellis.it

**Keywords:** MASLD, anthocyanins, cohort study

## Abstract

**Background**: MASLD is characterised by chronic inflammation and oxidative stress, which contribute to disease progression. Currently, no effective pharmacological treatment is available, and the first-line treatment remains lifestyle modification, including dietary changes and physical activity. This study aimed to assess the effect of dietary antioxidants, anthocyanins, on the risk of MASLD in a cohort from Southern Italy. **Methods**: The sample of this study comprised 1, 297 individuals aged between 54 and 64 years from a larger cohort, the NUTRIHEP study cohort. Data on anthocyanin intake were collected using a food-frequency questionnaire. MASLD is diagnosed when fatty liver disease is present in conjunction with at least one cardiometabolic risk factor. **Results**: Anthocyanin intake was inversely associated with MASLD risk. In Model b, adjusted for adjusted for age, sex, Fasting Glucose, Triglycerides, Diastolic Blood Pressure, Job, Alcohol consumption (g/day), daily energy intake, adherence to the Relative Mediterranean Diet (rMED), Available Carbohydrates, fibre intake, the third quartile (Q3) and the highest intake group (Q4) of anthocyanins showed a negative correlation with MASLD. Analysis of Anthocyanin intake as a continuous variable showed a modest negative association with MASLD risk (OR = 0.990, 95% CI 0.989–0.999), suggesting that higher anthocyanin intake may slightly lower the risk of MASLD. **Conclusions**: Our study highlights the protective effects of dietary anthocyanins against MASLD. These findings confirm the potential preventive role of dietary polyphenols in MASLD and identify anthocyanins as novel targets for intervention.

## 1. Introduction

Non-Alcoholic Fatty Liver Disease (NAFLD) is among the most common chronic liver conditions worldwide, affecting 25–45% of the general population [1]. It is often called a “silent epidemic” because one in four people are impacted. The prevalence increases to 60% among individuals with diabetes, as well as those who are obese or overweight [2]. The increasing prevalence of NAFLD in individuals with metabolic syndrome has led to the replacement of the term NAFLD with metabolic-associated steatotic liver disease (MASLD) [3]. MASLD is considered a more appropriate concept than NAFLD [4], given that steatosis is a hepatic manifestation of metabolic syndrome [4]. The diagnostic criteria for MASLD include the presence of hepatic steatosis and at least one of the following three features: overweight based on body mass index (BMI), type 2 diabetes mellitus (T2DM), and lean or normal weight with evidence of metabolic dysregulation [3]. The pathogenesis of fatty liver disease is complex and poorly understood. The key mechanisms driving MASLD progression include oxidative stress, chronic inflammation, and metabolic dysregulation, which contribute to hepatic steatosis, fibrosis, and insulin resistance [5]. A persistent inflammatory state compromises cellular homeostasis, promoting the development of a pro-oxidative state and persistence of the inflammatory state both locally and systemically [6]. For example, prolonged intestinal inflammation can impair the integrity of the intestinal epithelial barrier, facilitating the translocation of microbial products, such as lipopolysaccharides (LPS). The latter, through portal circulation, reaches the liver where it triggers an inflammatory response, leading to mitochondrial dysfunction and lipid buildup, which can contribute to steatosis [7]. An additional factor in the development and progression of MASLD is oxidative stress, caused by excessive production of reactive oxygen species (ROS) [8]. Although ROS are normally generated in small amounts during metabolism, their overproduction disturbs the balance between ROS creation and removal, as well as the repair of cellular damage [9]. These reactive molecules interact with lipids, proteins, and DNA, causing lipid peroxidation, protein oxidation, and DNA damage, which ultimately result in hepatocellular injury, inflammation, and fibrosis.

The cause of MASLD involves genetic and environmental factors, such as an unhealthy lifestyle [10]. Currently, there are no FDA-approved drug treatments for MASLD. As a result, lifestyle modifications—like increasing physical activity and adopting new dietary habits—are the primary approach [11]. Dietary elements and micronutrients, including flavonoids, are strongly linked to MASLD [12,13,14].

Flavonoids are natural food compounds found in fruits, vegetables, tea, and red wine. Several in vitro and animal studies have confirmed the potential of flavonoids to prevent and slow the progression of NAFLD [15]. They possess anti-inflammatory, antioxidant, and anti-metabolic properties that help mitigate oxidative stress and inflammation while improving lipid metabolism and insulin sensitivity [16]. Given these properties, there is growing interest in studying these dietary components to assess their potential for preventing and treating MASLD [17].

Flavonoids comprise various subclasses such as anthocyanins, isoflavones, flavones, flavanones, flavan-3-ols, and others. Earlier research mainly concentrated on anthocyanins, which exhibit a stronger protective effect against NASH compared to other flavonoid categories [18].

Anthocyanins are water-soluble flavonoids responsible for the color of fruits, which varies from red–orange to blue–violet color. The main dietary sources of anthocyanins are fruits, grains, and vegetables, such as blackcurrants, red raspberries, elderberries, chokeberries, strawberries, plums, pomegranates, blood oranges, beans, cabbage, and red onions. After ingestion, gut microbiota in the colon break down anthocyanins, generating metabolites like protocatechuic, ellagic, and vanillic acids. These metabolites appear to have stronger biological effects than the original anthocyanins or may possess other additional effects [19].

Overall, evidence suggests that regularly eating fruits high in anthocyanins is an effective approach to influence inflammatory and oxidative responses [20]. However, research indicates that the positive impacts of anthocyanins differ among ethnic groups, probably due to variations in lifestyle, dietary habits, and genetic makeup [21].

The Mediterranean Diet (MD) is a dietary regimen that ensures optimal anthocyanin intake. In the MD, red wine is a key source of beneficial polyphenols like anthocyanins. Since red grapes, and thus red wine, contain anthocyanins, they give the wine its red color [22]. It is characterised by a daily intake of vegetables, fruit and cereals and by regular but moderate consumption of wine, fish and dairy products, with extra virgin olive oil as the main source of fat for cooking and dressing. Therefore, it is primarily a plant-based diet rich in polyphenols, vitamins, and various other components that have positive effects on inflammatory and oxidative profiles [23]. A meta-analysis indicated that polyphenols, including anthocyanins and flavonoids, are the main contributors to the antioxidant activity of pomegranates. Pomegranate juice supplementation improves oxidative stress and inflammation by reducing the levels of IL-6 and TNF-α [24]. The beneficial effects of anthocyanins have been extensively demonstrated in vivo and in vitro. However, since their bioavailability is low, these effects are more noticeable in in vivo studies. The effects of anthocyanins are closely linked to their interactions with the gut microbiota [19]. It has been shown that blueberry anthocyanins improve liver lipid metabolism and liver steatosis by activating Takeda G protein-coupled receptor 5 (TGR5) and Farnesoid X Receptor (FXR) via the bile acid pathway [25]. Bile acids directly suppress the growth of Bacteroides and Lactobacilli in the gut, affecting the host’s production of inducible nitric oxide synthase (iNOS) [26].

This study aimed to assess the effects of dietary anthocyanins on MASLD risk in a cohort from Southern Italy.

## 2. Materials and Methods

### 2.1. Study Population

The NUTRIHEP cohort study started in 2005–2006, selecting a systematic random sample of individuals aged 18 or older from patient lists of Primary Care Physicians in Putignano (BA), Italy [27]. From 2015 to 2018, all participants were invited to the first follow-up, where 1426 individuals responded and completed the same standardized protocol used at baseline [28]. Written informed consent was obtained from all participants after providing detailed information about the use of their medical records. This study was a cross-sectional investigation focused exclusively on follow-up measurements and was approved by the Ethics Committee of the Ministry of Health (DDG-CE-792/2014) on 14 February 2014.

### 2.2. Data Collection

During follow-up visits, participants completed all assessments outlined in the study protocol. Trained physicians and/or nutritionists conducted structured interviews to gather sociodemographic, medical, and lifestyle information, including smoking history, dietary habits, education level, occupation, and marital status. Anthropometric measurements were taken with participants wearing only underwear and no shoes. Body weight was measured to the nearest 1 kg using an electronic scale (SECA), while height was measured to the nearest 1 cm with a wall-mounted stadiometer (SECA, Hamburg, Germany). Blood pressure (BP) was recorded following international guidelines [29,30], with the average of three readings calculated. Dietary habits were assessed through the validated European Prospective Investigation into Cancer and Nutrition (EPIC) Food Frequency Questionnaire (FFQ) [31,32], which participants completed independently and were verified by nutritionists before processing. The data was analyzed using a dedicated online tool to determine micro- and macronutrient intakes.

Biochemical testing included fasting serum glucose (FSG), insulin, Glycosylated Hemoglobin (HbA1c), triglycerides, total cholesterol, High-Density Lipoprotein Cholesterol (HDL-C), Aspartate Aminotransferase (AST), Alanine Aminotransferase (ALT), Alkaline Phosphatase (ALP), Gamma-Glutamyl Transferase (GGT), ferritin, and high-sensitivity C-reactive protein. All parameters were measured with a COBAS 8000 autoanalyzer (ROCHE Diagnostics SPA, Monza, Italy). Insulin resistance was estimated using the Homeostasis Model Assessment of insulin resistance (HOMA-IR) [33], calculated as: HOMA-IR = FSG (mg/dL) × fasting insulin (μIU/mL)/405. Liver steatosis was assessed via standard ultrasound using a Hitachi H21 Vision (Hitachi Medical Corporation, Tokyo, Japan) with a 3.5 MHz transducer. The hepatic fat content was scored semi-quantitatively based on liver echotexture, echo penetration, intrahepatic vessel visibility, and diaphragm differentiation [34].

Figure S1 shows the ultrasound reference chart used for grading steatosis.

### 2.3. Outcome Assessment

**Figure 1:**
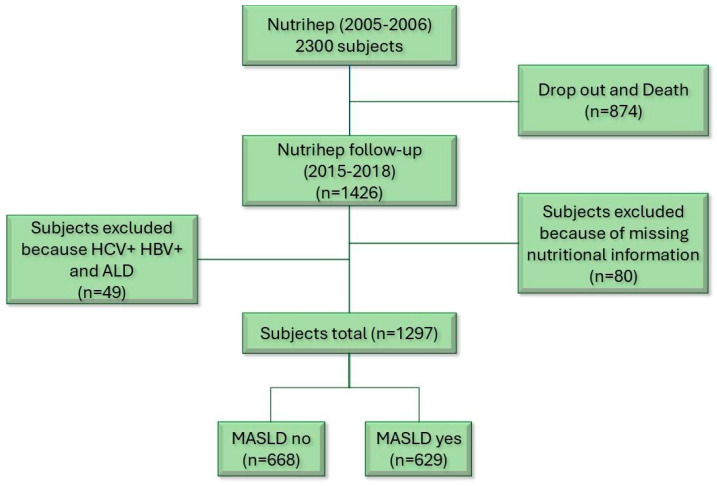
Flow Chart. ALD, alcohol-related liver disease; MASLD: Metabolic Dysfunction-Associated Steatotic Liver Disease.

MASLD is characterised by hepatic steatosis and at least one cardiometabolic risk factor: (1) BMI > 25 kg/m^2^ or waist circumference > 94 cm in men and >80 cm in women; (2) fasting glucose ≥100 mg/dL, 2 h post-load glucose ≥ 140 mg/dL, HbA1c ≥ 5.7%, or use of glucose-lowering medication; (3) blood pressure ≥ 130/85 mmHg or antihypertensive treatment; (4) triglycerides ≥ 150 mg/dL or lipid-lowering therapy; (5) HDL cholesterol < 40 mg/dL in men and <50 mg/dL in women, or targeted lipid-lowering therapy. In line with previous NAFLD criteria, MASLD diagnosis also requires limited alcohol intake, defined as <20–50 g/day for women and 30–60 g/day for men [35]. To avoid confounding, individuals with other liver diseases, such as MASLD with HCV or HBV co-infection, were excluded from the analysis (Figure 1).

### 2.4. Exposure Variable

The amounts of Anthocyanin (mg/day) for each food analyzed in this study (see Table 1) were sourced from the Phenol-Explorer database, which is available online at http://phenol-explorer.eu/ (accessed on 1 February 2026) [36].

### 2.5. Confounding Variables

Covariates were selected based on previous research and clinical and statistical considerations regarding their potential association with MASLD. After assessing collinearity, we included demographic and lifestyle factors, such as age, sex, Diastolic Blood Pressure, occupation, education, adherence to the Relative Mediterranean Diet (rMED), daily energy intake, Available Carbohydrates, fibre intake, as well as laboratory measurements, including fasting glucose and triglycerides.

BMI was excluded from the model because it caused multicollinearity, given that 542 (86.2%) of these patients had a BMI >25 (kg/m^2^).

### 2.6. Statistical Analysis

Differences between groups were assessed using the Wilcoxon test for continuous variables and the χ^2^ test for categorical variables. Logistic regression models were used to estimate odds ratios (ORs) and 95% confidence intervals (CIs), with MASLD as the outcome and anthocyanin intake as the predictor variable. Intakes were analysed as continuous variables and by quantiles. ORs indicate the association between exposure and outcome: OR = 1 indicates no association, OR >1 indicates an increased risk, and OR <1 indicates a protective effect [37].

Model *a* was adjusted for age and sex, model *b* was adjusted for age, sex, Fasting Glucose, Diastolic Blood Pressure, work, Alcohol consumption (g/day), while model c was adjusted for age, sex, Fasting Glucose, Triglycerides, Diastolic Blood Pressure, work, Education, Alcohol consumption (g/day), daily energy intake, adherence to the Relative Mediterranean Diet (rMED), Available Carbohydrates, and fibre intake.

Candidate confounders were first chosen based on existing literature and then refined using the Least Absolute Shrinkage and Selection Operator (LASSO) [38]. To identify multicollinearity, the Variance Inflation Factor (VIF) was employed, and variables with a VIF greater than 5 were eliminated [39].

Continuous variables were summarised as Mean ± Standard Deviations, while categorical variables were presented as frequencies and percentages. Two-tailed significance was set at *p* < 0.05 to test the null hypothesis of non-association. Analyses were performed using Stata 19 software (StataCorp 2025, College Station, TX, USA).

## 3. Results

Table 2 shows the characteristics of 1297 participants classified by MASLD status. Overall, 629 (48.50%) had MASLD, including 43.95% of women (327/744) and 54.61% of men (n = 302/553). Those with MASLD were generally older and exhibited higher rates of hypertension and hyperlipidemia compared to those without MASLD. They also had higher BMI (30.28 ± 4.97 kg/m^2^) and weight (79.58 ± 14.73 kg). Education levels were typically lower among the MASLD group: 423 participants had primary or secondary education, versus 242 in the non-MASLD group. Only 59 MASLD participants were university graduates, compared to 119 in the non-MASLD group. Blood parameters were elevated in participants with MASLD, with significant differences observed across multiple markers.

Compared with patients without MASLD, those with MASLD had significantly higher anthocyanin intake.

Table S1 details the characteristics of the 1297 participants by MASLD status and age group. This also indicates that older individuals tend to have a higher anthocyanin intake and are more likely to follow the Mediterranean diet.

We established a multivariate logistic regression model to explore the association between flavonoid subclasses and MASLD. The results showed that four flavonoid subclasses (flavonols, flavanones, flavones, and flavanols) were not significantly associated with the risk of MASLD (Supplementary Table S2). Anthocyanins showed a significant association after correction for covariates.

Anthocyanin consumption was negatively associated with MASLD risk (Table 3). In Model a, adjusted for age and sex, there was no statistically significant association with MASLD; however, a trend towards an inverse relationship was observed.

In Model b, adjusted for age, sex, fasting glucose, diastolic blood pressure, occupation, education, and alcohol consumption (g/day), the third quartile (Q3: 16.52–36.46 mg/day) and the highest intake group (Q4: 36.47–216.00 mg/day) of anthocyanins were negatively associated with MASLD (OR = 0.669, 95% CI 0.465–0.963 and OR = 0.643, 95% CI 0.417–0.992, respectively).

After further adjustments for age, sex, fasting glucose, triglycerides, diastolic blood pressure, work, education, alcohol consumption (g/day), daily energy intake, adherence to the Relative Mediterranean Diet (rMED), available carbohydrates, and fiber intake, model c showed that the third quartile (Q3: 16.52–36.46 mg/day) and the highest intake group (Q4: 36.47–216.00 mg/day) of anthocyanins were negatively associated with MASLD (OR = 0.640, 95% CI: 0.433–0.944 and OR = 0.626, 95% CI: 0.491–0.909, respectively), indicating a slight improvement in the estimates. (see Table 3).

In other words, consuming 16 to 216 mg of anthocyanins daily can decrease the risk of MASLD by 40%.

Analysis of Anthocyanin intake (model *c*) as a continuous variable showed a modest negative association with MASLD risk (OR = 0.990, 95% CI 0.989; 0.999, *p*-value: 0.037), suggesting that higher anthocyanin intake may slightly lower the MASLD risk. (Table 3).

## 4. Discussion

There is no doubt that MASLD is a significant global health issue and is likely to become a major public health concern in the future. Greater focus has been directed towards preventing MASLD through dietary and lifestyle modifications [40]. Our research explored how dietary anthocyanins affect a population in Southern Italy. Consistent with previous research [21], the risk reduction for MASLD appears to differ based on ethnicity and is affected by factors such as diet and eating habits. Until now, no studies have evaluated this outcome in a population similar to our study group.

In the study cohort, individuals with MASLD exhibited higher anthocyanin intakes. This appears to conflict with the existing literature and the known anti-inflammatory and antioxidant properties of flavonoids. Typically, individuals with MASLD tend to be obese or overweight and have metabolic issues more frequently than those without MASLD. A possible explanation for the increased anthocyanin intake is the higher consumption of fresh fruits, which are key sources of fructose and anthocyanins. Additionally, in our sample, participants with MASLD were older and more likely to follow the Mediterranean diet, resulting in a higher intake of anthocyanin-rich foods than those without MASLD.

Although our study cohort showed a higher intake of anthocyanin-rich foods in the MASLD group than in the non-MASLD group, the adjusted logistic models indicated an inverse association between MASLD and the intake of anthocyanin-rich foods. We began with a basic model adjusted for sex and age, then added variables such as fasting blood glucose, diastolic blood pressure, employment status, alcohol consumption (g/day) and total energy intake, and finally a more comprehensive model that incorporated age, sex, fasting blood glucose, triglycerides, diastolic blood pressure, occupation, education, alcohol consumption (g/day), daily energy intake, relative Mediterranean diet adherence (rMED) and carbohydrate and fibre intake. Logistic regression analysis demonstrated that higher anthocyanin intake was associated with a protective effect against MASLD.

Anthocyanins may lower the risk of MASLD through several mechanisms. Their proposed mode of action relates to their redox potential, allowing them to function as electron acceptors [41,42]. They counteract oxidative stress caused by oleic acid (OA-) by boosting antioxidant levels and promoting β-oxidation, which helps prevent mitochondrial dysfunction [43,44].

Another potential mechanism involves activating autophagy in tissues. When AMPK is phosphorylated, it triggers tissue autophagy processes that promote ‘cell renewal’ by removing damaged proteins and organelles caused by oxidative and inflammatory stress. This reduces inflammatory cytokines like TNF-α and IL-6, as well as lipid peroxidation [45,46]. Additionally, research indicates that anthocyanins can inhibit NF-κB and JNK/STAT3 pathways while increasing Nrf2 expression, which helps decrease inflammation and boost antioxidant defenses [44].

Some studies suggest that anthocyanins may have antihypertensive effects. This activity is believed to occur through several mechanisms, including modulating endothelial nitric oxide synthase (eNOS) expression, acting as antioxidants, inhibiting angiotensin-converting enzyme (ACE), and other pathways in endothelial cells. These effects may help reduce liver damage by improving vascular tone and hemodynamics, lowering blood pressure and portal pressure, which could be especially beneficial for patients with MASLD and hypertension [47].

The beneficial effects of anthocyanins on MASLD may partly result from weight reduction. A weight loss of 3–5% has been shown to improve steatosis, while a 7–10% loss can enhance inflammation, ballooning hepatocyte phenotype, and fibrosis in those with non-alcoholic steatohepatitis [48,49]. Khan et al. explain that this effect involves alterations in the mitogen-activated protein kinase (MAPK) and NF-κB stress signalling pathways, which promote cytoprotective and anti-inflammatory responses [50]. Some research indicates that anthocyanin-induced weight loss in MASLD is linked to changes in leptin and adiponectin levels, leading to decreased fat accumulation [51]. Additionally, other studies suggest that anthocyanins inhibit pancreatic lipase activity, reducing fat absorption in the intestine and, therefore, decreasing fat buildup [52].

Another potential benefit of anthocyanin consumption in MASLD is an improved lipid profile. Dyslipidemia, affecting 50% of individuals with MASLD, plays a role in its development [53]. A meta-analysis found that anthocyanin intake significantly lowered serum total cholesterol, triglycerides, and LDL levels, while raising HDL levels in people with dyslipidemia [54], likely by increasing fecal excretion of sterolic acids and inhibiting cholesterol synthesis—both effects induced by anthocyanins [55]. In our study, cholesterol and triglyceride levels were higher in the MASLD group despite their greater anthocyanin intake compared to the no-MASLD group. Nevertheless, although these levels were elevated compared to the no-MASLD group, they remained within normal ranges. Essentially, in our population, the primary metabolic alteration indicative of MASLD beyond liver steatosis was overweight.

Bartosz et al. reviewed how anthocyanins influence inflammation and oxidative stress, noting that their hepatoprotective effects do not act alone [56]. A decrease in intestinal inflammation correlates with reduced systemic levels of pro-inflammatory cytokines like TNF-α and IL-6, often accompanied by improved liver function indicators [57,58]. This might result from less LPS translocation into the portal circulation, which reduces activation of hepatic Toll-like receptors, NF-κB signalling, and the production of inflammatory mediators [59,60].

Certain plant-based foods contain nutritional components that are fermented by the gut microbiota in the colon, producing substances that affect inflammation, oxidative stress, and metabolic regulation [61]. Anthocyanins can modify the gut microbiota’s composition, increasing its diversity and abundance, and promoting the production of short-chain fatty acids (SCFA) [62]. Additionally, anthocyanins work synergistically with other bioactive compounds, amplifying their effects [63]. Ellagitannins, found in foods like cloudberries, blueberries, raspberries, and strawberries, are polyphenolic compounds present alongside anthocyanins [64]. When consumed, ellagitannins are broken down into ellagic acid (EA) in the stomach. Only a small portion is absorbed there, with most reaching the colon, where the microbiota converts it into urolithins, mainly urolithin A (UA) and urolithin B (UB) [65]. Both EA and urolithins have antioxidant and anti-inflammatory properties, which can help improve MASLD [61]. A study by Yin Qin et al. showed that Rubus corchorifolius L. fruit extract (RCE), rich in polyphenols including EA and anthocyanins, positively affects mitochondrial damage and oxidative stress in the liver, reducing MASLD [66].

### Strength and Limitations

Our study examined the role of anthocyanins in the development and prevention of MASLD, a topic for which human research is limited [18]. Another strength is the study sample’s characteristics, as no previous study has examined this topic in a population such as ours.

However, this study had some limitations. Information on dietary habits was collected using the EPIC questionnaire, a nationally validated food-frequency questionnaire. However, it does not include certain foods rich in anthocyanins, such as cherries, which are widely consumed by the population during certain seasons. Another potential limitation is the reliance on self-reported dietary data; however, to address this, each FFQ was reviewed by our dietitian upon receipt of the questionnaire. Another important limitation is the study’s design. Since it was cross-sectional, it cannot establish causality, and further research is necessary to confirm our findings. Additionally, we did not directly measure physical activity, a key lifestyle factor closely linked to MASLD risk and progression, which could serve as an unmeasured confounder [67]. Although we controlled for various sociodemographic, dietary, and biochemical variables, residual confounding may still exist [68].

## 5. Conclusions

Our study results highlight the protective role of dietary anthocyanins against MASLD. These findings support the idea that dietary polyphenols could help prevent MASLD and suggest that anthocyanins are a new target for intervention. Nonetheless, additional research is necessary to validate these results and identify the specific dietary component levels needed for protection.

## Figures and Tables

**Table 1:** Mean daily intake of anthocyanin (mg/day) from food by MASLD, as reported in the EPIC food frequency questionnaire. NUTRIHEP cohort 2015–2018.

	All Sample	MASLD No	MASLD Yes
Anthocyanin (mg/day)	Mean (SD)	Mean (SD)	Mean (SD)
Total Intake	31.57 (30.67)	28.16 (27.59)	35.20 (33.27)
Red Wine	15.01 (38.95)	12.71 (47.91)	17.45 (26.11)
White Wine	0.004 (0.01)	0.00 (0.01)	0.01 (0.02)
Red Onion	0.07 (0.15)	0.06 (0.14)	0.09 (0.17)
Aubergine	0.04 (0.04)	0.04 (0.04)	0.04 (0.04)
Peach	0.09 (0.07)	0.09 (0.07)	0.09 (0.07)
Plum	5.11 (6.19)	4.93 (5.83)	5.30 (6.55)
Strawberry	7.25 (12.54)	7.16 (11.87)	7.35 (13.24)
Grape Black	4.72 (5.13)	4.57 (4.99)	4.87 (5.28)

**Table 2:** Characteristics of participants by MASLD status, NUTRIHEP cohort, 2015–2018.

Variables ^a^	Whole Sample ^b^	MASLD		
		No	Yes	*p*-Value ^c^
N (%)	1297	668 (51.50)	629 (48.50)	
**Exposure variables:**				
Anthocyanidins (mg/day)	31.57 (30.67)	28.16 (27.59)	35.19 (33.27)	<0.001
Flavonoid intake:				
Total Flavonoids (mg/day)	174.45 (153.33)	160.89 (178.50)	188.86 (119.46)	0.001
Flavones (mg/day)	12.39 (13.29)	12.59 (13.27)	12.17 (13.33)	0.58
Flavanones	17.33 (14.74)	16.39 (16.04)	18.33 (13.15)	0.018
Flavonols	11.52 (12.88)	10.48 (15.25)	12.61 (9.64)	0.003
Flavanols	105.64 (97.57)	96.44 (112.69)	115.42 (77.28)	<0.001
**Demographic and lifestyle characteristics**				
Age (years)	54.33 (14.34)	49.24 (13.80)	59.74 (12.86)	<0.001
Sex (%)				
Female	744 (57.36)	417 (56.05)	327 (43.95)	<0.001
Male	553 (42.64)	251 (45.39)	302 (54.61)	
rMED	8.04 (2.55)	7.91 (2.54)	8.18 (2.56)	0.05
rMED Score (%)				
Low	365 (28.14)	196 (53.70)	169 (46.30)	0.46
Moderate	705 (54.36)	362 (51.35)	343 (48.65)	
High	227 (17.50)	110 (48.46)	117 (51.54)	
Alcohol intake (g/day)	10.58 (12.72)	10.74 (13.41)	10.42 (11.96)	0.66
SFA (g/day)	251.73 (102.15)	258.52 (99.01)	244.53 (104.97)	0.014
AC (g/day)	25.40 (11.32)	26.43 (11.58)	24.31 (10.93)	<0.001
Fibre Intake (g/day)	22.63 (8.41)	22.69 (7.99)	22.55 (8.83)	0.76
Kcal (day)	2056.26 (750.22)	2100.33 (724.88)	2009.46 (774.05)	0.029
Smoker (%)				
Never/Former	1137 (87.73)	587 (51.63)	550 (48.37)	0.87
Current	159 (12.27)	81 (50.94)	78 (49.06)	
Marital Status (%)				
Single	181 (13.96)	115 (63.54)	66 (36.46)	<0.001
Married or living together	1034 (79.72)	519 (50.19)	515 (49.81)	
Separated or Divorced	28 (2.16)	20 (71.43)	8 (28.57)	
Widow/er	54 (4.16)	14 (25.93)	40 (74.07)	
Education (%)				
Primary school	282 (21.74)	71 (25.18)	211 (74.82)	<0.001
Secondary school	383 (29.53)	171 (44.65)	212 (55.35)	
High School	460 (35.47)	307 (66.74)	153 (33.26)	
Graduate	172 (13.26)	119 (69.19)	53 (30.81)	
Work (%)				
Managers & Professionals	102 (7.86)	57 (55.88)	45 (44.12)	<0.001
Craft, Agricultural, and Sales Workers	469 (36.16)	285 (60.77)	184 (39.23)	
Elementary Occupations	185 (14.26)	93 (50.27)	92 (49.73)	
Housewife	141 (10.87)	74 (52.48)	67 (47.52)	
Pensioners	325 (25.06)	110 (33.85)	215 (66.15)	
Unemployed	75 (5.78)	49 (65.33)	26 (34.67)	
Family income assessment (%)				
insufficient	27 (2.08)	10 (37.04)	17 (62.96)	0.025
just sufficient	167 (12.88)	81 (48.50)	86 (51.50)	
sufficient	1019 (78.57)	521 (51.13)	498 (48.87)	
more than sufficient	64 (4.93)	44 (68.75)	20 (31.25)	
good	20 (1.54)	12 (60.00)	8 (40.00)	
**Anthropometric and clinical parameters**				
BMI (kg/m^2^)	27.58 (5.05)	25.04 (3.59)	30.28 (4.97)	<0.001
Weight (kg)	72.93 (14.87)	66.66 (12.02)	79.58 (14.73)	<0.001
Waist (cm)	90.45 (13.46)	83.04 (10.38)	98.32 (11.79)	<0.001
SBP (mmHg)	120.93 (15.81)	115.64 (15.35)	126.52 (14.30)	<0.001
DBP (mmHg)	77.68 (8.00)	75.69 (7.88)	79.78 (7.58)	<0.001
Hypertension (%)				
No	847 (68.75)	517 (61.04)	330 (38.96)	<0.001
Yes	385 (31.25)	115 (29.87)	270 (70.13)	
Dyslipidemia (%)				
No	1047 (85.05)	561 (53.58)	486 (46.42)	<0.001
Yes	184 (14.95)	71 (38.59)	113 (61.41)	
Diabetes (%)				
No	1148 (93.18)	620 (54.01)	528 (45.99)	<0.001
Yes	84 (6.82)	12 (14.29)	72 (85.71)	
**Blood Tests**				
HbA1c (mmol/mol)	38.07 (6.87)	36.59 (5.05)	39.64 (8.09)	<0.001
Glucose (mg/dL)	95.34 (17.34)	90.13 (10.54)	100.89 (21.06)	<0.001
HOMA-IR	1.89 (1.88)	1.33 (0.90)	2.43 (2.38)	<0.001
ALT (U/L)	22.20 (16.21)	19.70 (8.27)	24.86 (21.37)	<0.001
GGT (U/L)	17.58 (13.46)	14.80 (7.67)	20.54 (17.16)	<0.001
AST (U/L)	21.74 (10.87)	20.70 (5.94)	22.85 (14.29)	<0.001
ALP (U/L)	52.98 (16.10)	50.10 (15.56)	56.04 (16.11)	<0.001
TG (mg/dL)	98.41 (69.23)	80.73 (58.55)	117.22 (74.60)	<0.001
TC (mg/dL)	191.35 (35.36)	188.90 (33.06)	193.96 (37.50)	0.010
HDL-C (mg/dL)	50.79 (12.59)	53.18 (12.80)	48.24 (11.85)	<0.001
C-reactive protein (mg/dL)	0.26 (0.55)	0.21 (0.52)	0.31 (0.58)	<0.001

Notes: ^a^ Values are expressed as mean ± standard deviation. ^b^ Percentages were calculated by column; otherwise, percentages were calculated by row. ^c^ Continuous variables were compared using the Wilcoxon rank-sum test, and categorical variables were compared using the χ^2^ test. Abbreviations: MASLD: Metabolic Dysfunction-Associated Steatotic Liver Disease; rMED: Relative Mediterranean Diet; Saturated Fatty Acids (SFA); Available Carbohydrates (AC), BMI: Body Mass Index; SBP: Systolic Blood Pressure; DBP: Diastolic Blood Pressure; HbA1c: Glycosylated Hemoglobin; HOMA: Homeostasis Model Assessment; ALT: Alanine Aminotransferase; GGT: Gamma-Glutamyl Transferase; AST: Aspartate Aminotransferase; TG: Triglycerides; TC: Total Cholesterol; HDL-C: High-Density Lipoprotein Cholesterol; ALP: Alkaline Phosphatase.

**Table 3:** Logistic regression analysis of the association between anthocyanin intake and MASLD.

MASLD	OR *	*p*-Value	95% CI
Model *a*			
Anthocyanidins quartiles (mg/day)			
<7.51	1.000		
7.57–16.51	0.853	0.349	0.611, 1.190
16.52–36.46	0.801	0.201	0.570, 1.125
36.47–216.00	0.805	0.230	0.564, 1.148
Anthocyanidins total intake (mg/day)	0.999	0.626	0.995; 1.003
Model *b*			
Anthocyanidins quartiles (mg/day)	1.000		
<7.51	1.000		
7.57–16.51	0.844	0.342	0.596, 1.196
16.52–36.46	0.669	0.030	0.465, 0.963
36.47–216.00	0.643	0.046	0.417, 0.992
Anthocyanidins total intake (mg/day)	0.996	0.234	0.990, 1.002
Model *c*			
Anthocyanidins quartiles (mg/day)			
<7.51	1.000		
7.57–16.51	0.818	0.279	0.569, 1.177
16.52–36.46	0.640	0.025	0.433, 0.944
36.47–216.00	0.626	0.049	0.391, 0.909
Anthocyanidins total intake (mg/day)	0.990	0.037	0.989; 0.999

Note: The daily intake of anthocyanidins is presented as continuous variables and as quantiles. * No MASLD: reference category. Models: Model *a* was adjusted for age and sex, model *b* was adjusted for age, sex, Fasting Glucose, Diastolic Blood Pressure, Work, Alcohol consumption (g/day), while model *c* was adjusted for age, sex, Fasting Glucose, Triglycerides, Diastolic Blood Pressure, Work, Education, Alcohol consumption (g/day), daily energy intake, adherence to the Relative Mediterranean Diet (rMED), Available Carbohydrates, fibre intake. Abbreviations: MASLD: Metabolic Dysfunction-Associated Steatotic Liver Disease.

## Data Availability

The data supporting the findings of this study are openly available at https://doi.org/10.6084/m9.figshare.32134897 (accessed on 2 May 2026).

## Supplementary Materials

The following supporting information can be downloaded at https://www.mdpi.com/article/10.3390/antiox15070802/s1. Figure S1. Ultrasound Scan Board; Table S1. Characteristics of participants by MASLD status and age categories; Table S2. Logistic regression results for the association between intake of Flavanols, Flavonones, Flavones, and Flavonols and the risk of MASLD.
