# Supplementary material for: Dietary Anthocyanin Intake and Risk of Metabolic Dysfunction-Associated Steatotic Liver Disease: Results from the NUTRIHEP Study
Source: Antioxidants (Basel). 2026 Jun 26;15(7):802. doi: 10.3390/antiox15070802 (PMC13404332; doi:10.3390/antiox15070802)
Supplement: Supplementary file 1 [file antioxidants-15-00802-s001.zip › antioxidants-4340645-supplementary.pdf]

Liver Ultrasound

**Dimensions**	Normal	Increase	Reduced
**Margins**	Regular	Irregulars	
**Ecostructure**	Homogeneous Normoechogenic	Uneven	Hyperechogenic

Evaluation of Hepatic Steatosis

**Contrast between liver parenchyma (P. EPA) and renal parenchyma (P. REN)**	**Deep beam penetration ultrasonic**	**Sharpness of the vascular structures, particularly particularly the veins**
Homogeneous echo level and contrast between P. EPA and P. REN not evident (0)	Hepatic parenchyma clearly visible from the surface to the diaphragm (0)	Vascular structures clearly visible (0)
Slight discrepancy in echogenicity hepatic-renal (1)	Presence of opacity of the deeper parts of the liver or failure to visualize the diaphragm (1)	Loss of echoes of the vascular structures (1)
Wide discrepancy between hepatic and renal (2)	Presenza di opacità delle parti più profonde del fegato e mancata visualizzazione del diaframma (2)	Vascular structures not clearly visible (2)
**Steatosis score:** Steatosis Absent (0); Mild Steatosis (1-2); Moderate Steatosis (3-5); Severe Steatosis (6)		

Figure S1. Ultrasound Scan Board.

Table S1. Characteristics of participants by MASLD status and age categories, NUTRIHEP Study 2015–2018.

	Age Categories						
	<30<br>Mean (SD)	30-40<br>Mean (SD)	41-50<br>Mean (SD)	51-60<br>Mean (SD)	61-70<br>Mean (SD)	71-80<br>Mean (SD)	>80<br>Mean (SD)
MASLD No							
N	36	175	164	128	109	43	13
AnthoCyanins (mg/day)	19.28 (17.92)	19.62 (18.72)	25.32 (27.42)	29.20 (25.89)	39.96 (32.27)	44.44 (38.35)	40.38 (25.72)
rMED	7.61 (2.37)	7.64 (2.62)	7.67 (2.45)	8.16 (2.71)	8.31 (2.43)	8.26 (2.12)	8.23 (3.09)
BMI	23.61 (3.17)	24.15 (3.70)	24.66 (3.13)	25.63 (3.79)	26.32 (3.25)	25.86 (3.57)	26.43 (4.39)
Alcohol consumption (g/day)	15.43 (13.74)	14.08 (15.89)	10.89 (14.16)	7.80 (10.52)	8.68 (11.51)	8.64 (8.21)	3.85 (6.24)
Daily energy intake	2,359.60 (710.51)	2,226.28 (760.21)	2,104.02 (720.38)	2,079.83 (642.85)	1,935.49 (689.31)	1,915.21 (795.25)	1,836.81 (747.97)
MASLD Yes							
N	6	53	82	143	218	105	22
AnthoCyanins	15.83 (8.77)	19.21 (20.97)	26.19 (26.26)	35.86 (31.72)	39.77 (36.58)	40.37 (34.77)	38.28 (35.74)
rMED	6.33 (2.73)	7.83 (2.49)	7.59 (2.61)	8.24 (2.45)	8.33 (2.71)	8.63 (2.30)	7.82 (2.42)
BMI	25.59 (2.94)	30.30 (5.77)	30.21 (5.32)	29.90 (4.79)	30.82 (4.91)	30.17 (4.83)	29.56 (3.62)
Alcohol consumption (g/day)	21.99 (16.12)	12.76 (14.47)	10.17 (11.63)	12.32 (12.98)	9.12 (10.91)	8.65 (10.90)	11.61 (10.11)
Daily energy intake	2,409.28 (875.95)	1,986.87 (663.23)	2,207.86 (742.88)	2,218.01 (808.49)	1,912.94 (789.88)	1,816.85 (713.94)	1,735.42 (517.51)

MASLD: Metabolic Dysfunction-Associated Steatotic Liver Disease; rMED: Relative Mediterranean Diet; BMI: Body Mass Index

Table S2 The logistic regression analysis results of the association between intake of Flavanols, Flavonones, Flavones, and Flavonols and the risk of MASLD.

Variables	Quartile 1	Quartile 2	Quartile 3	Quartile 4
Flavanols				
Range (mg/day)	<49.88	49.88 - 87.85	87.86 - 137.69	137.70 - 345.32
Model a [OR (95% CI) (P-value)]	Referent	0.853 [0.615, 1.224 (0.419)]	0.868 [0.615, 1.224 (0.419)]	0.972 [0.675, 1.399 (0.878)]
Model b [OR (95% CI) (P-value)]	Referent	0.766 [0.526; 1.116 (0.165)]	0.743 [0.498; 1.110 (0.147)]	0.892 [0.536; 1.486 (0.661)]
Flavanones				
Range (mg/day)	<6.83	6.83 - 16.74	16.75 - 23.66	23.67 - 67.50
Model a [OR (95% CI) (P-value)]	Referent	0.821 [0.585, 1.152 (0.254)]	1.076 [0.766, 1.513 (0.672)]	1.026 [0.727, 1.446 (0.885)]
Model b [OR (95% CI) (P-value)]	Referent	0.842 [0.582, 1.219 (0.363)]	1.114 [0.766, 1.620 (0.573)]	1.105 [0.736, 1.658 (0.631)]
Flavones				
Range (mg/day)	<1.36	1.36 - 7.61	7.62 – 19.17	19.18 - 54.00
Model a [OR (95% CI) (P-value)]	Referent	0.807 [0.577, 1.127 (0.208)]	0.781 [0.560, 1.091 (0.147)]	0.834 [0.597, 1.165 (0.287)]
Model b [OR (95% CI) (P-value)]	Referent	0.755 [0.526, 1.085 (0.129)]	0.700 [0.487, 1.008 (0.055)]	0.804 [0.550, 1.176 (0.260)]
Flavonols				
Range (mg/day)	<5.13	5.13 - 8.56	8.57 – 14.62	14.63 – 63.85
Model a [OR (95% CI) (P-value)]	Referent	1.104 [0.790, 1.544 (0.562)]	0.928 [0.662, 1.301 (0.663)]	1.073 [0.751, 1.534 (0.697)]
Model b [OR (95% CI) (P-value)]	Referent	1.086 [0.743, 1.589 (0.669)]	0.720 [0.476, 1.089 (0.120)]	0.782 [0.465, 1.316 (0.355)]

Note: OR, Odds Ratio; 95% CI, 95% Confidence Interval. Models: Model a: adjusted for age, gender; Model b: adjusted for age, gender, occupation, education, family income, smoking, adherence to the Relative Mediterranean Diet, daily energy intake, Saturated Fatty Acids, Available Carbohydrates, fibre intake, Aspartate Aminotransferase, fasting glucose, triglycerides, and platelets. Abbreviations: MASLD: Metabolic Dysfunction-Associated Steatotic Liver Disease;
